# Pharmacokinetic, pharmacodynamic, and clinical aspects of ovulation induction agents: A review of the literature

**DOI:** 10.4274/jtgga.2016.0107

**Published:** 2017-03-01

**Authors:** Serkan Kahyaoğlu, Bülent Yılmaz, Ahmet Zeki Işık

**Affiliations:** 1 Department of Obstetrics and Gynecology, Zekai Tahir Burak Women’s Health Training and Research Hospital, Ankara, Turkey; 2 Department of Obstetrics and Gynecology, İzmir Katip Çelebi University Faculty of Medicine, Tepecik Training and Research Hospital, IVF Unit, İzmir, Turkey; 3 Assisted Reproductive Technologies Unit, Medical Park Hospital, İzmir, Turkey

**Keywords:** Pharmacokinetic, pharmacodynamic, ovulation induction, infertility

## Abstract

Controlled ovarian hyperstimulation is a key step for successful outcomes of assisted reproductive technique cycle outcomes. Many medications are available, which are commonly useed solely or in combination to achieve multiple follicular development. Pharmacokinetic, pharmacodynamic, and clinical information of ovulation induction drugs deserve to be elucidated for every individual patient before commencing infertility treatment. New concepts and new treatment protocols are introduced as ovulation physiology is understood by infertility specialists. Increasing treatment success by minimizing aderse effects is a milestone of all ovarian stimulation protocols that use these novel interventions. Achievement of a satisfactory cycle outcome includes retrieval of sufficient oocytes, a single clinical pregnancy, and avoidance of ovarian hyperstimulation syndrome. In this review, we evaluate the current literature to determine the most reliable and relevant information about the most used ovulation induction drugs.

## INTRODUCTION

Pharmacokinetics is the study of drug metabolism in the body according to the rates of three processes: absorption, distribution, and elimination. Pharmacodynamics is the study of the mechanism of action by which drugs exert their pharmacologic effects; the binding of a drug to its target receptor or enzyme followed by a signal transduction pathway by which the receptor activates second messenger molecules, and finally the description of intracellular processes altered by the impact of the drug are components of the pharmacokinetics. Pharmacogenetics and pharmacogenomics are the study of the role of genetic inheritance in individual variation to drug response. Administration of a drug to different individuals can result with different clinical results based on the pharmacogenomic variability among individuals rather than pharmacokinetics. Individualisation of drug therapy can be tailored in the future by using pharmacogenomic information.

Recently, infertility became a relatively common public health problem because of the increased prevalance of advanced childbearing age of women. Ovulation induction treatment accompanied by artificial insemination or assisted reproduction are commonly used in infertile women. Pharmacokinetic, pharmacodynamic, and pharmacogenetic aspects of commonly used infertility drugs should be known to improve cycle outcomes. In this review, we aimed to discuss these clinical issues by evaluation of the current published literature regarding ovulation induction agents.

The ovulation induction agents that are commonly used during infertility treatment are shown in the Table 1.

## 1. ANTIESTROGENS (SELECTIVE ESTROGEN RECEPTOR MODULATORS, AROMATASE INHIBITORS)

### a. Selective estrogen receptor modulators

Estrogen reseptor modulators exert partial agonist and antagonist effects according to the tissue estrogen receptor content and estrogen availability level. Selective estrogen receptor modulators (SERMs) act by inhibiting the negative feedback effect of circulating estrogen on the hypothalamic pituitary unit (1). Clomiphene citrate (CC), tamoxifen, and raloxifene are three commonly used SERMs in women’s health care.

CC is well absorbed from the gastrointestinal tract when administered orally. The commonly used daily dosage of CC is between 50-150 mg. Lower than 50 mg doses can be needed for the hyperresponder patient group, especially patients with polycystic ovary syndrome (PCOS). Although rarely needed, higher than 150 mg doses increase the antagonistic effect of CC on the endometrium and cervix, which is not warranted. CC is metabolized by hepatic transformation and excreted by feces which increases its bioavailability. High binding capacity to plasma proteins, entering enterohepatic cycle and accumulation in fatty tissues incerases the elimination half life of CC (5 days). Tamoxifen has a slightly higher elimination half life than CC (7 days). CC is exactly a weak estrogen agonist and a moderate estrogen antagonistic molecule. SERMs act on estrogen receptor containing tissues such as the hypothalamus, pituitary, ovary, endometrium, vagina, and cervix by competing with estrogen and decreasing the intracellulary estrogen receptor content. CC contains two isomeric forms, both of which include different clinical efficacy. Zuclomiphene is the less potent form with long elimination half time, which still exists in the body during early pregnancy achieved by utilization of CC for ovulation induction. Enclomiphene is the more potent form with short elimination half time which mainly exerts the clinical effect of CC following oral administration. CC is a category X drug but congenital anomaly rates are similar to the normal population. Enclomiphene is the more potent form with a short half life.

In two observational studies, ovulation and pregnancy rates seemed to be improved for patients with PCOS who were treated with tamoxifen following CC failure (2, 3). According to a Cochrane review conducted by Brown et al. (4), pregnancy rate, ovulation rate, miscarriage rate, live birth rate, and ongoing pregancy rate were similar between ovulation induction with CC and tamoxifen.

### b. Aromatase inhibitors

Anastrazole and letrozole are nonsteroid competetive inhibitors of aromatase. These drugs have been developed for treatment of locally-advanced and metastatic breast cancer of postmenopausal women. Following oral administration, their elimination half time is 2 days. Hot flushes, nausea, headache, vaginal bleeding, and backache are adverse effects. The estrogen suppresion effect of aromatase inhibitors (AI) are dose dependant. The hypothalamo-pituitary-ovarian axis remains intact during ovulation induction treatment and this advantage results with monofollicular ovulation and lower multiple pregnancy rates. Absence of hostile antiestrogenic effect of CC on endometrium and cervix is another benefit of AIs. In a prospective randomized trial conducted by Diamond et al. (5), ovarian stimulation using letrozole resulted in a significantly lower rate of multiple pregnancy accompanied by a lower rate of live birth when compared with gonadotropin, but not when compared with CC treatment among women with unexplained infertility. Legro et al. (6) conducted another prospective randomized trial and they concluded that when compared with clomiphene, higher live birth and ovulation rates were achieved with ovulation induction using letrozole among infertile women with PCOS. Roque et al. (7) performed a systematic review based on randomized controlled trials comparing cycle outcomes of CC and letrozole among patients with PCOS. A statistically significant increase in the live birth and pregnancy rate was detected in the letrozole group when compared with CC use [relative risk (RR)=1.55 and RR=1.38, respectively]. Ovulation, miscarriage, and multiple pregnancy rates between the two groups were found similar. The authors concluded that regarding live birth and pregnancy rates, ovulation induction using letrozole results with better cycle outcomes when compared with CC in patients with PCOS (7). Letrozole’s pharmacodynamic beneficial effects result with higher pregnancy rates when compared with CC. Letrozole has shorter elimination half time (45 hours) than CC. Accumulation of CC within the body results with extended depletion of estrogen receptors accompanied by hostile effects on estrogen sensitive genital tissues. Letrozole increases the biosynthesis of endometrial receptivity markers such as integrins. In 2005, an oral presentation at an American Society of Reproductive Medicine meeting increased concerns regarding congenital malformation and teratogenicity risks of letrozole. This presentation has since been critisized because of the design of the study and the lack of publication in a peer-reviewed journal. Contrarily, cardiac and congenital abnormality rates of pregnancies achieved with CC have been found increased in some studies (8, 9). Tulandi et al. (10) performed a multicenter study comparing the neonatal outcome of 514 letrozole pregnancies with 297 CC pregnancies in 2006 and they concluded that congenital malformation and chromosomal abnormality rates of letrozole and CC were similar (2.4% vs. 4.8%, respectively). In addition, the cardiac anomaly rate of CC was significantly higher than letrozole (1.8% vs. 0.2%, respectively; p=0.02).

## 2. METFORMIN

Metformin is a biguanide oral antidiabetic medication that increases the sensitivity of insulin receptors in peripheral cells. Adding metformin to treatment cycle protocols for increasing pregnancy rates among patients with PCOS is a matter of debate. In a systematic review, Palomba et al. (11) concluded that infertile patients with PCOS treated with gonadotrophins for in vitro fertilization (IVF)/intracytoplasmic sperm injection (ICSI) cycles, implantation rates seemed improved but pregnancy or live birth rates did not increase by using metformin despite lower rates of ovarian hyperstimulation syndrome (OHSS) and miscarriage (11, 12). Specific phenotypes and features of patients with PCOS who will benefit from metformin should be defined before liberally advising metformin to all patients with PCOS. Longer than 3 weeks administration of metformin has been found to decrease miscarriage rates [odds ratio (OR) 0.41, 95% confidence interval: (0.21 to 0.78), p=0.0086]. In a Cochrane database review by Tso et al. (13) in which the clinical effects of metformin treatment before and during IVF or ICSI in women with PCOS were evaluated, the authors concluded that despite significantly beneficial effects for OHSS prevention, no conclusive evidence has been detected for improved live birth rates by using metformin treatment before or during assisted reproductive technique (ART) cycles. Unlike Palomba et al. (11), they emphasized that the use of this insulin-sensitising agent increased clinical pregnancy rates without exerting any beneficial effect on abortus rate, retrieved oocyte number, total gonadotropin dose, stimulation time, fertilization and cycle cancellation rate (13).

## 3. GONADOTROPINS

Follicle-stimulating hormone (FSH), luteinizing hormone (LH), human chorionic gonadotropin (hCG), and thyroid stimulating hormone are heterodimer glycoprotein hormones including alfa and beta subunits. Alfa subunits of these hormones are made up of same 92 aminoacides. The beta subunit is responsible for the biologic specificity of the hormone. The serum elimination half times of these hormones are relatively short except hCG. Although the beta subunits of LH and hCG have the same rate of 80%, the plasma elimination half time of hCG is 10 times higher than LH. C terminal peptides and sialic acid residues containing 31 amino acids cause this paharmacocinetic difference. The bioavailability of recombinant FSH (rFSH) and recombinant LH (rLH) is around 70% and 60% following subcutaneous administration, respectively (14, 15, 16). No pharmacokinetic interactions occur between rFSH and rLH when administered simultaneously. The serum elimination half time of rFSH and rLH is around 24 and 10-12 hours, respectively. Steady state plasma levels are achieved after 3-4 days following repeated rFSH injections. Among patients whose endogenous hypothalamopituitary axis have been suppressed, rFSH alone can efficiently achieve folliculogenesis and also steroidogenesis despite low serum LH levels. Different clinical responses to the same FSH medication doses are caused by FSH receptor polymorphism, also called pharmacogenetics, rather than pharmacokinetic actions of the drug (14, 15, 16, 17).

Batch-to-batch inconsistency, foreign proteins, and unpredictable clinical eficiency are major drawbacks of urine-derived gonadotropins. In a prospective randomized multicenter study, Frydman et al. (18) compared rFSH with urinary FSH according to ART cycle outcomes. Achievement of a higher number of oocytes with lower total doses and shorter stimulation times, rFSH was found more potent than urinary FSH. However, the increased oocyte numbers were not reflected by increased pregnancy rates for rFSH against urinary FSH.

Stimulation of folliculogenesis in the treatment of infertility has been traditionally conducted by using gonadotropins extracted from the urine of postmenopausal women. Urine-derived products consist of a mixture of gonadotropins with unpredictable clinical efficiencies and biologically active mediators such as binding proteins, growth factors, and prion proteins. The variation of exact amount of gonadotropins in human menopausal gonadotropin (hMG) preparations results with diverse effects on gonads during ovulation induction. The hCG content and hMG product increases parallel to the increasing purity of the drug to standardise the biologic activity. hCG is secreted by the embryo and placenta and physiologically supports implantation and pregnancy. The receptor binding affinity of hCG is 2 times higher than LH. LH has a shorter serum elimination half life than hCG (23 vs. 32-33 hours, respectively). hCG accumulates in the body significantly and causes LH receptor downregulation unlike LH itself. Six to 8 IU of LH is biologically equivalent to 1 IU of hCG, which demonstrates the potency of hCG over LH. Controversy has not been resolved as to whether r-hLH or hCG should be used for ART to increase cycle outcomes (19).

In a prospective observational study, Requena et al. (20) compared endocrine profile of oocyte donors stimulated with FSH plus rLH (2/1 in ratio) or hMG. Although retrieved oocyte numbers following treatment with recombinant gonadotropins were higher than urinary gonadotropins (hMG) (16.5 vs. 11.8; p=0.049), harvested metaphase II ooocyte numbers were higher by using urinary gonadotropins (hMG) (71.2% vs. 80.6%; p=0.003). When serum steroid hormone levels (estradiol, progesterone, testosterone and androstenedione) were evaluated on the day of triggering and cycle day 6, slightly elevated levels were detected in the recombinant gonadotropins when compared with the urinary gonadotropins. Comparison of intrafollicular levels of steroid hormones were found statistically insignificant between the two protocols and ongoing pregnancy rates were also similar (46.1% vs. 46.1%) (20).

In the Menopur in GnRH Antagonist Cycles with Single Embryo Transfer (MEGASET) trial, Devroey et al. (21) evaluated the safety and efficacy of rFSH and highly purified menotropin (hphMG) for controlled ovarian hyperstimulation in GnRH antagonist cycles with mandatory single blastocyst transfer. Although higher oocyte numbers were achieved with rFSH against hMG, similar MII oocyte numbers were harvested. The authors concluded that despite the significant discrepancy in pharmacodynamic effects, highly purified hMG was found have a similar effect as rFSH in GnRH antagonist cycles with mandatory single blastocyst transfer based on clinical pregnancy rates of both fresh and freeze thaw cycle transfers of day 5 embryos (21).

Recently, a gonadotropin preparation that includes rFSH and rLH 2/1 in ratio was commercially developed. Dosing studies performed on hypogonadotropic hypogonadism patients that evaluted the clinical eficacy of this new drug revealed that 75 IU of LH were sufficient for optimal folliculogenesis (22). Some studies in the literature have demonstrated the nourishing effects on cycle outcomes and ovarian response rates of addition of LH activity to stimulation regimens in certain groups of patients. Women aged older than 35 years, those with diminished ovarian reserve, and women with LH receptor polymorphisms are theoretical candidates for this approach. Adding LH to the treatment protocol activates theca cells to produce more androgens, which are eventually converted to estrogens in granulosa cells to increase the estrogenic milieu within the ovarian follicle and also oocyte quality (23).

Pacchiarotti et al. (24) conducted a prospective randomised trial to compare IVF outcomes in ovarian stimulation protocols with recombinant FSH plus recombinant LH (2/1 in ratio) versus hMG. Treatment with rFSH plus rLH or with hMG was found to produce the same results in terms of implantation rates, pregnancy rates, and embryo quality. Although a statistical difference in oocyte quality, with a better quality in the hMG group was detected, this difference was levelled because of the total number of oocytes retrieved, which was higher in the rFSH plus rLH group, thus the total number of MII oocytes was similar in both groups at the expense of higher OHSS rates for rFSH plus RLH group. The reduction of the amount of FSH used in the hMG group also led to lower cost of the IVF cycle (24).

Bosch (25) published a review article regarding the pharmacologic characteristics and clinical applications of rFSH plus rLH (2/1 in ratio). Although the 2:1 combination of r-hFSH and r-hLH seems to be an optimum ovulation induction regimen regarding safety and clinical efficacy in patients with hypogonatrophic hypogonadism, use of this drug combination in ovarian stimulation for IVF remains controversial because the target population that may receive a benefit from this combination therapy is not well defined. Patients needing >3000 IU rFSH during COH, patients showing plateau on follicular growth, and those with inadequate response after 7 days r-FSH have been suggested as candidates for adding rLH to stimulation regimens based on previous studies (25).

In a systematic review and meta-analysis, Lehert et al. (26) suggested that in poor responders, r-hLH supplementation of r-hFSH compared with rhFSH alone may result in significantly higher oocyte numbers, clinical pregnancy rates, and ongoing pregnancy rates. Based on this entity, Humaidan et al. (27) are currently conducting a randomized controlled multicenter trial to explore the possible advantages of a fixed-dose combination of r-FSH plus r-LH over r-FSH monotherapy in patients with poor ovarian response (POR) according to the definition determined in the European Society of Human Reproduction and Embryology (ESHRE) Bologna criteria.

### Long-acting gonadotropins

Corifollitropin alfa is a long acting recombinant FSH, which acts for 7 days following administration to support folliculogenesis. Although pharmacodynamic actions of long-acting rFSH is the same as with rFSH, the serum elimination half time of long-acting rFSH is 65 hours, which is twice that of rFSH. Dose finding studies revealed that patients weighing above and below 60 kg, 100 µg and 150 µg long-acting rFSH are recommended for clinical efficiency (28). In a Cochrane database meta-analysis, Pouwer et al. (29) revealed that although the use of a medium dose (150 to 180 μg) of long-acting rFSH seemed to be a safe and equally effective treatment option when compared with daily rFSH in women with unexplained subfertility, reduced live birth rate in women receiving a low dose (60 to 120 μg) of long-acting rFSH compared with daily rFSH was also observed.

The safety and effectiveness of long-acting FSH for use in hyper- or poor responders and in women with all causes of subfertility is an area of current research. In a systematic review and meta-analysis including 4 randomized trials, Mahmoud Youssef et al. (30) concluded that corifollitropin alfa in combination with daily GnRH antagonist seemed to be an alternative for daily rFSH injections in view of efficiency and safety profile among normoresponder patients undergoing controlled ovarian hyperstimulation in IVF/ICSI treatment cycles.

## 4. RECOMBINANT HUMAN CHORIONIC GONADOTROPIN VERSUS URINARY HUMAN CHORIONIC GONADOTROPIN

hCG is used for final maturation of oocytes during ART cycles. Urine-derived hCG has some disadvantages compared with recombinant hCG (rhCG) such as batch-to-batch inconsistency, uncontrolled source, and unpredictable biologic activity. Chang et al. (31) compared the efficacy and safety of 250 µg and 500 µg of rhCG with 10 000 IU of urinary hCG (uhCG) in ART in a randomized controlled prospective study. As the primary end point of the study, total harvested oocyte numbers were similar for both groups. Based on the results of this study, rhCG was found effective and tolerable in terms of induction of final follicular maturation and luteinization for women undergoing ART procedures. Youssef et al. (32, 33) performed two consecutive Cochrane metaanalyses to assess the safety and efficacy of subcutaneous rhCG and high-dose rLH compared with intramuscular uhCG for inducing final oocyte maturation triggering in IVF and ICSI cycles and they concluded that equivalent pregnancy rates and OHSS incidences were found between rhCG or rhLH and uhCG when used for final follicular maturation in IVF. According to these findings, the authors recommended using uhCG as the best selection for final oocyte maturation triggering in IVF and ICSI treatment cycles.

## 5. GONADOTROPIN-RELEASING HORMONE AGONIST TRIGGER FOR FINAL OOCYTE MATURATION

hCG has been used as a surrogate for midcycle LH peak to induce final oocte maturation before oocyte retrieval in ART. The relatively long elimination half time of hCG obtains a luteotrophic effect during the luteal phase, but also increases the OHSS risk. Despite obtaining a stimulus for final oocyte maturation, ovulation triggering with hCG has no beneficial effect on endometrial receptivity and oocyte quality when compared with spontaneous ovulation (34). The FSH surge accompanies the LH surge during physiologic ovulation that triggers natural cycles. This midcycle surge of FSH is thought to promote nuclear maturation of the oocyte, cumulus cell accumulation, and LH receptor formation on granulosa cells. When GnRH antagonists were introduced to the market, the use of GnRH agonists for final oocyte maturation came into consideration again.

Pioneer studies in this field resulted with disappointment regarding low pregnancy rates and high abortion rates of IVF-ET cycles triggered with GnRH agonists (35). Modifications of luteal phase support solved this clinical problem and nowadays GnRh agonists are more frequently used for final maturation, especially for patients with increased OHSS risk. Although GnRH agonist trigger strategy seems to decrease OHSS risk with satisfactory pregnancy rates by using modified luteal phase support; early OHSS can still occur even when embryo transfer is deferred (36, 37, 38). Oocyte donors, high responser patients, patients who demand fertility preservation, and also normal responder patients are suggested as the target groups for GnRH agonist trigger. During the luteal phase of ART cycles triggered with GnRH agonists, the relatively shortstanding LH surge and central inhibition of gonadotropin secretion due to supraphysiological serum estradiol levels causes depletion of LH support, which is needed by the corpora lutea to enhance implantation by secretion of progesterone and also many other implantation favoring mediators. Although luteal estradiol supplementation is not needed for ART cycles triggered with hCG, this intervention is strongly recommended until the 7^th^ gestational week during ART cycles triggered with GnRH agonists (38). Humaidan et al. (36) suggested administering 1500 IU hCG intramuscularly during oocyte retrieval procedures when GnRH agonists have been used for final oocyte maturation of GnRH antagonist cycles. This intervention has dramatically lowered abortion rates and boosted the pregnancy rates for this group of patients.

Different doses of different GnRh agonists have been successfully used for final oocyte maturation in the literature. Youssef et al. (39) performed a Cochrane metaanalysis to evaluate the differences between GnRH agonists and HCG in terms of safety and effectiveness for triggering final oocyte maturation in IVF-ICSI among women undergoing a GnRH antagonist protocol. Unlike the Humaidan group, the authors concluded that when GnRH agonists were used for final oocyte maturation in fresh autologous cycles, lower live birth rates, lower ongoing pregnancy rates, and a higher rates of early miscarriage were achieved. Youssef et al. (39) recommended the use of GnRH agonists as an oocyte maturation trigger for women who are spared for fresh transfers, who are oocyte donors, and who demand to freeze autologous oocytes for fertility preservation. Recently, Engmann et al. (40) reviewed the advantages and potential drawbacks of GnRH agonist triggering by performing a strengths, weaknesses, opportunities and threats (SWOT) analysis. Based on this analysis modality, the authors recommended intensive luteal support with transdermal oestradiol and intramuscular progesterone alone if peak serum oestradiol is 4000 or more pg/mL after GnRHa triggering or dual triggering with GnRH agonist and hCG 1000 IU if peak serum oestradiol is less than 4000 pg/mL. The recommendations of the same group based on the follicle number were as follows: administration of hCG 1500 IU 35 h after GnRH agonist trigger if there are less than 25 follicles ≥11 mm on the day of ovulation trigger, or freeze all oocytes or embryos if there are over 25 follicles (40).

## 6. GONADOTROPIN-RELEASING HORMONE ANTAGONIST (SHORT) VERSUS GONADOTROPIN-RELEASING HORMONE AGONIST (LONG) PROTOCOL

Al-Inany et al. (41) conducted a Cochrane metaanalysis for comparing these mostly used COH protocols. They investigated the safety and effectiveness of GnRH antagonists by comparison with the long protocol of GnRH agonists for ovarian stimulation in ART cycles. In this review, the authors concluded that when compared with long GnRH agonist protocols, the antagonist protocol was associated with a wide decrease in OHSS rates and similar live birth rates (41, 42). The same group recently conducted a Cochrane systematic review and similar live birth rates were observed between GnRH antagonist and long GnRH agonist protocols. When compared with GnRH agonists, GnRH antagonist-based protocols lowered the incidence of all OHSS severity grades (OR 0.61). The miscarriage rates were found similar between these two protocols. The cycle cancellation rate following POR to ovulation induction was higher in women who received GnRH antagonist protocols compared with GnRH agonist protocols (OR 1.32) (43). Based on these results, GnRH antagonist protocols seem to be the best and safest protocol for patients with high baseline OHSS risk. Contrarily, GnRH agonist protocols result with higher oocyte yield than GnRH antagonist protocols among poor responder patients. Sunkara et al. (44) performed a randomized controlled study among poor responders undergoing IVF treatment. The number of oocytes retrieved was significantly higher with long GnRH agonists compared with short agonist regimens (4.42±3.06 vs. 2.71±1.60) and similar between long agonist and antagonist regimens (4.42±3.06 vs. 3.30±2.91). Total gonadotropin dose and duration of stimulation were significantly higher using long agonist regimens compared with short agonist and antagonist regimens. The ongoing pregnancy rates were 16.2% with antagonist protocols and 8.1% with long and short agonist protocols (p=0.48). Based on these results, the authors concluded that long GnRH agonist and antagonist regimens can be a better selection as ovulation induction regimens for poor responders, whereas the short agonist regimen seems to be a less effective treatment strategy because fewer oocytes are retrieved (44). Al Inany et al. conducted a Cochrane systematic review including 73 RCTs, with 12 212 participants, comparing GnRH antagonist to long-course GnRH agonist protocols. Although the quality of the selected studies for this systematic review was moderate, the use of GnRH antagonist was found associated with a substantial reduction in OHSS without reducing the likelihood of achieving live birth when compared with long-course GnRH agonist protocols (43).

In conclusion, rational use and administration of ovulation induction drugs necessitate evaluation of pharmacokinetic, pharmacodynamic, and clinical aspects of each individual medication based on pharmacologic and clinical evidence. This clinical practice will eventually increase the success of ovulation induction protocols performed for infertility treatment and decrease the health threatening risks that arise from the treatment burden.

## Figures and Tables

**Table 1:**
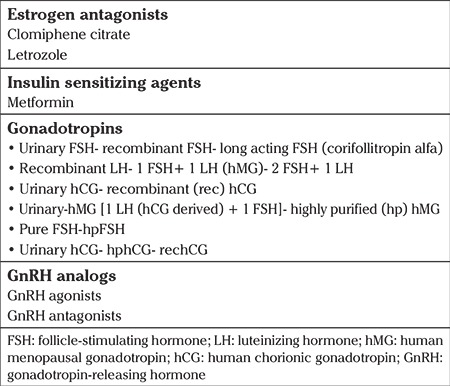
Commonly used ovulation induction agents
